# Meta-analysis of nonsteroidal anti-inflammatory drug intake and prostate cancer risk

**DOI:** 10.1186/1477-7819-12-304

**Published:** 2014-10-05

**Authors:** Xiao Wang, Yi-wei Lin, Jian Wu, Yi Zhu, Xiang-lai Xu, Xin Xu, Zhen Liang, Zheng-hui Hu, Shi-qi Li, Xiang-yi Zheng, Li-ping Xie

**Affiliations:** Department of Urology, The First Affiliated Hospital, School of Medicine, Zhejiang University, 79 Qingchun Road, Hangzhou, 310003 Zhejiang Province People’s Republic of China

**Keywords:** Etiology, Meta-analysis, NSAIDs, Prostate cancer

## Abstract

**Background:**

Epidemiological studies of the association between nonsteroidal anti-inflammatory drug (NSAID) intake and the risk of prostate cancer still remain controversial. Therefore, we conducted a meta-analysis to evaluate the potential association between NSAID intake and prostate cancer risk.

**Methods:**

Eligible studies were retrieved by both computerized searches and reviews of references. Subgroup analyses on country and design of study were also performed. Random or fixed-effect models were used to pool estimates of odds ratios (ORs) with 95% confidence intervals (CIs).

**Results:**

We observed that the intake of aspirin was associated with a marginally decreased risk of prostate cancer (OR =0.95, 95% CI =0.93 to 0.98). A similar result was found between nonaspirin NSAIDs and prostate cancer risk (OR =0.94, 95% CI =0.90 to 0.98). However, a positive relation between all-NSAID intake and prostate cancer risk was observed (OR =1.18, 95% CI =1.15 to 1.22).

**Conclusions:**

We observed a marginally inverse correlation between the intake of aspirin and prostate cancer risk. On the contrary, a positive relationship between all-NSAID intake and prostate cancer was detected. Further research needs to be conducted to better clarify potential biological mechanisms.

## Background

Prostate cancer is generally accepted as the most common type of cancer among men and ranks as the second cause of cancer-specific death in the whole world [1]. In the USA, approximately 238,590 men were diagnosed with prostate cancer, of which 12% were expected to die in 2013 [1]. Epidemiologic studies illustrated that both environmental and genetic alterations are well-established risk factors of prostate cancer [2, 3]. However, this could hardly explain the different incidences of prostate cancer in different countries [1, 4]. Therefore, daily lifestyle could play an important role in carcinogenesis.

Previous research demonstrated that nonsteroidal anti-inflammatory drugs (NSAIDs) could reduce the risk of several types of cancer, including colorectal cancer, esophageal cancer, and breast cancer [5–7]. Laboratory research has consistently illustrated that inflammation plays an essential role in prostate carcinogenesis [8]. It is generally accepted that cyclooxygenase enzymes, which are the targets of most NSAIDs, are involved in prostate carcinogenesis. A previous review, focusing on the potential relationship between NSAID intake and prostate cancer risk, showed that NSAIDs could prevent prostate cancer from developing into advanced disease, suggesting that NSAIDs could have a potentially protective effect on prostate cancer [9].

Nevertheless, epidemiological studies of the association between NSAID intake and the risk of prostate cancer still remained controversial. A previous meta-analysis in 2010 (including articles published before 2008) indicated that aspirin and other types of NSAID had a marginally, but not conclusively, protective effect on prostate cancer [10]. In addition, multicenter and large-sample studies were performed in the last 5 years, which provided us with more reliable statistics.

Therefore, we conducted a meta-analysis to evaluate both the strength and consistency of the potential association between NSAID intake and prostate cancer risk. Stratified analyses were also conducted on possible variables.

## Methods

### Literature search

**Figure 1:**
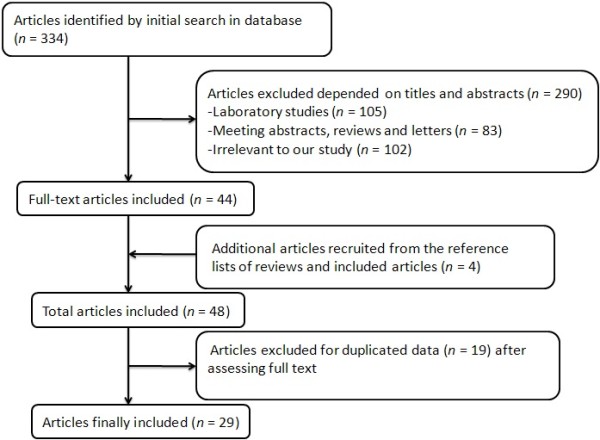
**Article selection.**

To obtain an overall view of NSAID intake and prostate cancer risk, we performed a comprehensive and systematic searching strategy. We searched for publications updated to October 2013 using PubMed, the Web of Science, and Cochrane Library. We selected the keyword search ‘(“Anti-Inflammatory Agents, Non-Steroidal” [Mesh] OR “Aspirin” [Mesh]) AND “Prostatic Neoplasms” [Mesh]’ to identify publications. Additional publications were assessed either by cited references in the recruited articles or reported meta-analyses on NSAID intake and prostate cancer risk. Each retrieved publication was evaluated for the following criteria: (1) case-control or cohort study assessing the potential correlation between NSAIDs intake and prostate cancer risk; (2) exact data in both case and control groups should be identified; (3) articles published before October 2013 written in English; (4) results including odds ratios (ORs) or relative risk and 95% confidence intervals (95% CIs), or supplying sufficient information to calculate them. Studies with insufficient or overlapping data were excluded. Figure 1 illustrates the process of determining and selecting articles.

### Data extraction

Data extracted from the articles included the name of the first author, year of publication, study period, country, type of NSAID, design of study, information resource, and odds ratio comparing the highest level of NSAID intake with the lowest. Given that prostate cancer is a relatively rare disease, the relative risk was considered to be the same as the OR. Therefore, we chose OR as the result to assess the potential relationship between NSAID intake and prostate cancer risk. Investigators were divided into two groups, and extracted data from all the potentially qualifying publications simultaneously, to avoid omissions or mistakes. Discussions were conducted whenever necessary.

### Statistical analysis

A fixed-effects model using the method of Mantel and Haenszel [11] was selected to provide pooled estimation of the correlation between NSAID intake and prostate cancer risk when no heterogeneity was detected; otherwise, the random-effect model of the method of DerSiomonian and Laird [12] was selected for summarization.

We chose OR and 95% CI to evaluate the strength of the relationship between NSAID intake (all NSAIDs, aspirin, and nonaspirin NSAIDs, respectively) and prostate cancer risk (prostate cancer and advanced prostate cancer, respectively). Advanced prostate cancer was defined as Gleason score ≥8, clinical stage ≥ T_2_C, or prostate-specific antigen ≥10 ng/ml. Stratified analyses were conducted by country (USA or other countries) and study design (cohort study or case-control).

We used the quantified *Q* test [12] and *I*^2^ test [13] to assess the extent of heterogeneity across eligible studies. Publication bias was evaluated by Egger’s [14] and Begg’s [15] tests. The statistical significance level was set at 0.05.

Sensitivity analyses were also conducted to evaluate the effect of each study on the overall estimate.

All statistical analyses were conducted using STATA version 11 (StataCorp, College Station, Texas, USA).

## Results

### Description of the meta-analysis

A total of 334 articles were found when the mentioned keywords were used. After a closer examination, 309 articles were excluded based on titles and abstracts, while 4 articles were recruited from the reference lists of reviews and included articles [16–19]. Figure 1 illustrates the detailed process of identifying and selecting articles. Finally, we identified a total of 29 articles on NSAID intake and prostate cancer risk that were published between 1989 and 2013 [16–44].

Among the included articles, 15 were cohort studies [16–20, 22, 24, 28–30, 33, 36, 37, 39, 40], and 14 were case-control ones [21, 23, 25–27, 31, 32, 34, 35, 38, 41–44]. Sixteen studies were conducted in the USA [16–18, 20, 22, 24, 25, 28, 30, 31, 37, 39–43], while the remaining 13 were performed in other countries [19, 21, 23, 26, 27, 29, 32–36, 38, 44]. Eleven studies were associated with all-NSAID intake [19, 20, 26–28, 32–34, 38, 42, 44], 24 with aspirin [16–18, 21–33, 36–41, 43, 44], and 14 with nonaspirin NSAIDs [23, 25–28, 30, 32, 35, 36, 38–41, 44]. Eleven studies were concerned about advanced prostate cancer [18, 24, 26–28, 30, 37–39, 41, 43]. Information was collected from either databases or questionnaires. Detailed characteristics of the eligible studies are listed in Table 1.

**Table 1:** **Characteristics of the included studies on nonsteroidal anti-inflammatory drugs intake and prostate cancer**

First author	Published year	Study design	Country	Drugs	Study period	Information resource
Paganini-Hill [16]	1989	Cohort	USA	Aspirin	1981 to 1988	Questionnaire
Schreinemachers [22]	1994	Cohort	USA	Aspirin	1971 to 1987	Questionnaire
Neugut [31]	1998	Case-control	USA	Aspirin	1984 to 1986	Questionnaire
Norrish [38]	1998	Case-control	New Zealand	Aspirin, NA-NSAIDs, NSAIDs	1996	Questionnaire
Nelson [25]	2000	Case-control	USA	Aspirin, NA-NSAIDs	1992 to 1995	Questionnaire
Langman [34]	2000	Case-control	UK	NSAIDs	1993 to 1995	Database
Leitzmann [18]	2002	Cohort	USA	Aspirin	1986 to 1998	Questionnaire
Roberts [20]	2002	Cohort	USA	NSAIDs	1990 to 1996	Questionnaire
Habel [24]	2002	Cohort	USA	Aspirin	1964 to 1996	Questionnaire
Irani [35]	2002	Case-control	France	NA-NSAIDs	1999 to 2000	Questionnaire
Sorensen [19]	2003	Cohort	Denmark	NSAIDs	1989 to 1995	Database
Friis [29]	2003	Cohort	Denmark	Aspirin	1989 to 1997	Database
Perron [33]	2003	Cohort	Canada	Aspirin, NSAIDs	1993 to 1996	Database
Ratnasinghe [17]	2004	Cohort	USA	Aspirin	1976 to 1992	Questionnaire
García Rodríguez [36]	2004	Cohort	UK	Aspirin, NA-NSAIDs	1995 to 2001	Database
Jacobs [28]	2005	Cohort	USA	Aspirin, NA-NSAIDs, NSAIDs	1992 to 2001	Questionnaire
Platz [40]	2005	Cohort	USA	Aspirin, NA-NSAIDs	1980 to 2004	Questionnaire
Bosetti [21]	2006	Case-control	Italy	Aspirin	1991 to 2002	Questionnaire
Dasgupta [23]	2006	Case-control	Canada	Aspirin, NA-NSAIDs	1999 to 2002	Database
Mahmud [26]	2006	Case-control	Canada	Aspirin, NA-NSAIDs, NSAIDs	1999 to 2003	Questionnaire
Menezes [41]	2006	Case-control	USA	Aspirin, NA-NSAIDs	1982 to 1998	Questionnaire
Murad [32]	2010	Case-control	UK	Aspirin, NA-NSAIDs, NSAIDs	2001 to 2008	Database
Brasky [39]	2010	Cohort	USA	Aspirin, NA-NSAIDs	2000 to 2007	Questionnaire
Coogan [42]	2010	Case-control	USA	NSAIDs	1992 to 2008	Questionnaire
Salinas [43]	2010	Case-control	USA	Aspirin	2002 to 2005	Questionnaire
Dhillon [37]	2011	Cohort	USA	Aspirin	1988 to 2006	Questionnaire
Mahmud [44]	2011	Case-control	Canada	Aspirin, NA-NSAIDs, NSAIDs	1985 to 2000	Questionnaire
Shebl [30]	2012	Cohort	USA	Aspirin, NA-NSAIDs	1993 to 2009	Questionnaire
Veitonmaki [27]	2013	Case-control	Finland	Aspirin, NA-NSAIDs, NSAIDs	1995 to 2002	Database

NA-NSAIDs, nonaspirin nonsteroidal anti-inflammatory drugs; NSAIDs, total nonsteroidal anti-inflammatory drugs.

### Intake of aspirin and prostate cancer

**Figure 2:**
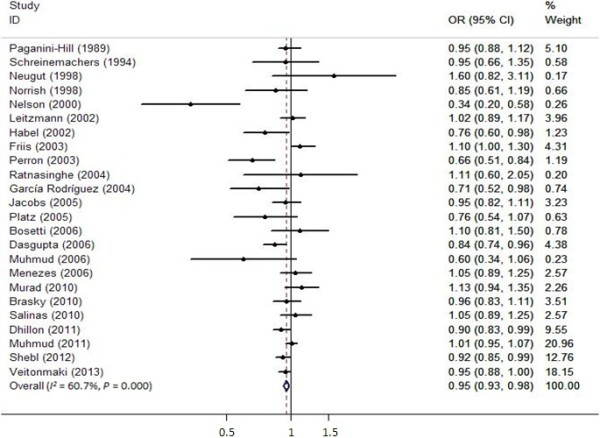
**Forest plots depicting the risk estimates from included studies on the association between the intake of aspirin and prostate cancer risk.** CI, confidence interval; OR, odds ratio.

Of the 24 studies concerned with aspirin intake, we observed that the intake of aspirin was associated with a marginally decreased risk of prostate cancer (OR =0.95, 95% CI =0.93 to 0.98) (Figure 2). A statistical heterogeneity was detected, therefore, random-effect analysis was performed (*I*^2^ = 60.7%, *P* >0.001). The risk of advanced prostate cancer was lower than that of prostate cancer (OR =0.89, 95% CI =0.82 to 0.96). The fixed-effect analysis was conducted and no significant heterogeneity was found (*I*^2^ = 4.5%, *P* =0.4).

Begg’s and Egger’s tests were performed to evaluate publication bias among the included studies; no such bias was found by either Begg’s or Egger’s test (*P* =0.503, *P* =0.160, respectively).

For stratified analysis, the summarized OR estimates indicated that the intake of aspirin was associated with a decreased risk of prostate cancer in the USA (OR =0.937, 95% CI =0.900 to 0.975). However, no such association was detected in other countries (OR =0.969, 95% CI =0.934 to 1.006). When subgroup analysis was performed by study design, we observed a marginally protective effect of aspirin on prostate cancer in the cohort group (OR =0.930, 95% CI =0.894 to 0.967). Nevertheless, no such effect was found in the case-control group (OR =0.976, 95% CI =0.940 to 1.013). Detailed data are given in Table 2.

**Table 2:** **Stratified pooled odds ratios and 95% confidence intervals of NSAIDs intake and prostate cancer**

Subgroup	Number of studies	Odds ratio (95% confidence interval)	Heterogeneity	
			***P***	***I*** ^2^(%)
**Aspirin**				
USA	14	0.937 (0.900, 0.975)	0.012	52.0
Other countries	10	0.969 (0.934, 1.006)	0.000	70.0
Cohort studies	13	0.930 (0.894, 0.967)	0.025	48.6
Case-control studies	11	0.976 (0.940, 1.013)	0.000	69.0
**NSAIDs**				
USA	3	0.922 (0.836, 1.016)	0.011	77.7
Other countries	8	1.212 (1.174, 1.251)	0.000	92.7
Cohort studies	4	1.022 (0.938, 1.113)	0.000	90.2
Case-control studies	7	1.205 (1.167, 1.245)	0.000	93.3
**NA-NSAIDs**				
USA	6	1.009 (0.927, 1.099)	0.807	0.0
Other countries	8	0.915 (0.869, 0.964)	0.001	70.3
Cohort studies	5	0.992 (0.909, 1.083)	0.629	0.0
Case-control studies	9	0.922 (0.876, 0.971)	0.002	67.9

### All-NSAID intake and prostate cancer

A total of 11 studies were associated with all-NSAID intake and prostate cancer. Compared with previous meta-analyses, we observed a positive relationship between all-NSAID intake and prostate cancer risk, owing to new research (OR =1.18, 95% CI =1.15 to 1.22). A random-effect analysis was conducted, owing to significantly statistical heterogeneity (*I*^2^ = 92.4%, *P* >0.001). Similar results were found between all-NSAID intake and advanced prostate cancer (OR =1.43, 95% CI =1.32 to 1.56, *I*^2^ = 92.8%, *P* >0.001). No publication bias was detected when Begg’s and Egger’s tests were conducted (*P* =0.312, *P* =0.190, respectively).

In the subgroup analysis, the pooled OR estimates suggested that no direct relationship between all-NSAID intake and prostate cancer risk was observed in the USA (OR =0.922, 95% CI =0.836 to 1.016). Notably, we detected a positive correlation between all-NSAID intake and prostate cancer risk (OR =1.212, 95% CI =1.174 to 1.251). When stratified analysis was conducted by study design, we did not observe a protective effect on prostate cancer in the cohort group (OR =1.022, 95% CI =0.938 to 1.113). However, in case-control groups, all-NSAID intake could be a potential risk factor in prostate carcinogenesis (OR =1.205, 95% CI =1.167 to 1.245). Detailed data are illustrated in Table 2.

### Nonaspirin NSAID intake and prostate cancer

**Figure 3:**
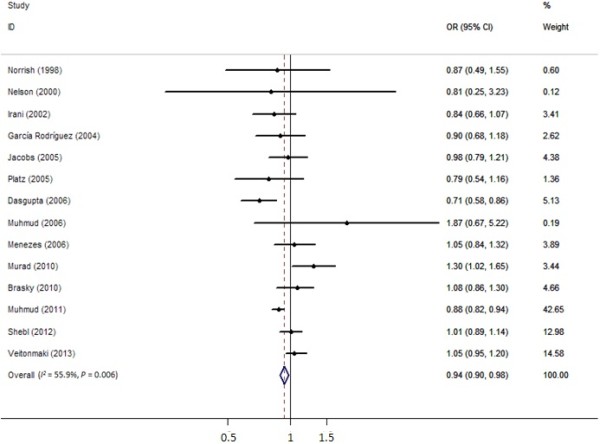
**Forest plots depicting the risk estimates from included studies on the association between nonaspirin NSAID intake and prostate cancer risk.** CI, confidence interval; OR, odds ratio.

Among the 14 articles related to nonaspirin NSAIDs and prostate cancer risk, we observed a marginally inverse relation (OR =0.94, 95% CI =0.90 to 0.98) (Figure 3). A significantly statistical heterogeneity was observed, thus random-effect analysis was performed (*I*^2^ = 55.9%, *P* >0.01). Nevertheless, an opposite relation was detected between nonaspirin NSAID intake and advanced prostate cancer (OR =1.25, 95% CI =1.13 to 1.38, *I*^2^ = 65.8%, *P* >0.01).

Begg’s and Egger’s tests were conducted to assess for publication bias among the included articles; no such bias was detected by either Begg’s or Egger’s test (*P* =0.956, *P* =0.500, respectively).

In the stratified analysis, the summarized OR estimates showed that nonaspirin NSAIDs were associated with a decreased risk of prostate cancer in other countries except the USA (OR =0.915, 95% CI =0.869 to 0.964). However, we failed to find a similar relation in the USA (OR =1.009, 95% CI =0.927 to 1.099). When stratified analysis was performed on study design, we observed a marginally protective effect on prostate cancer in the case-control group (OR =0.922, 95% CI =0.876 to 0.971). Nevertheless, no such result was detected in the cohort group (OR =0.992, 95% CI =0.909 to 1.083). Detailed data are illustrated in Table 2.

### Sensitivity analysis

Simultaneously, sensitivity analyses were conducted to evaluate the effect of each study on the overall estimate by sequentially excluding each study in turn. In our meta-analysis, we found that probably no study could affect the summary of risk estimate (data not shown).

## Discussion

Nonsteroidal anti-inflammatory drugs are one of the most commonly used medicine types in the whole world. Studies showed that daily nonsteroidal anti-inflammatory drug intake could be a non-invasive, economical, and valuable method of preventing cancer. Previous research illustrated that inflammation was involved in the process of prostate carcinogenesis [45]. Evidence demonstrated that cyclooxygenase enzymes, the targets of most NSAIDs, played a critical role in the development of prostate cancer [8]. Further studies verified that NSAIDs could inhibit the development and progression of prostate cancer, which meant that NSAIDs might be effective in the prevention of prostate cancer [9]. Nevertheless, conflicting results were obtained by different research centers. Therefore, we performed a meta-analysis to evaluate both the strength and consistency of the potential association between NSAID intake and prostate cancer risk.

We observed that the intake of aspirin was associated with a marginally decreased risk of prostate cancer (OR =0.95, 95% CI =0.93 to 0.98). The risk of advanced prostate cancer was less than that of prostate cancer (OR =0.89, 95% CI =0.82 to 0.96). This indicated that the intake of aspirin showed a marginally inverse correlation with prostate cancer risk. The results of the 24 studies included in our analysis were heterogeneous, probably resulting from differences in country and study design. To further illustrate the relationship between the intake of aspirin and prostate cancer risk, subgroup analyses were conducted. The summarized OR estimates illustrated that the intake of aspirin was associated with a decreased risk of prostate cancer while separately analyzed by country and design of study. In the stratified analysis by country, we found that an inversed correlation between the intake of aspirin and prostate cancer risk in the USA (OR =0.937, 95% CI =0.900 to 0.975). Conversely, no such association was observed in other countries (OR =0.969, 95% CI =0.934 to 1.006). Additionally, we found that none of the studies analyzing the association between the intake of aspirin and prostate cancer risk was conducted in Asian countries, probably because of different daily habits in those countries; this aspect requires further research.

A total of 11 studies were associated with all-NSAID intake and prostate cancer risk. We found a positive relation between all-NSAID intake and prostate cancer risk, owing to more recent research, compared with previous meta-analyses (OR =1.18, 95% CI =1.15 to 1.22). Similar results were found between all-NSAID intake and advanced prostate cancer (OR =1.43, 95% CI =1.32 to 1.56). To explain the phenomenon, we took a deeper look at the included studies. One study conducted in Finland illustrated a significantly elevated prostate cancer risk of total and advanced prostate cancer among all-NSAID users [27]. The association was not dose-dependent in the study, indicating that it could result from systematic differences between users of prescription NSAIDs and users of non-prescription NSAIDs. Furthermore, people who take prescribed NSAIDs are possibly frequent users of other health services, such as cancer screening, including testing for prostate-specific antigen. This could bring about a positive detection bias.

Meanwhile, among the 14 articles associated with nonaspirin NSAIDs and prostate cancer risk, we observed a marginally inverse correlation between them (OR =0.94, 95% CI =0.90 to 0.98). Nevertheless, an opposite correlation was detected between nonaspirin NSAIDs intake and advanced prostate cancer (OR =1.25, 95% CI =1.13 to 1.38).

### Our study could have limitations in several ways

Firstly, although no publication bias was detected in our analysis, using both Begg’s and Egger’s tests, the strategy of selection of published studies in English only and the exclusion of studies without sufficient information could bring about possible publication bias, which remains an alternative explanation of our results. Furthermore, our search was restricted to published articles, which could also cause potential bias to affect our findings.

Secondly, both case-control and cohort studies with different populations, drug intakes, and outcomes were included in our analysis. Considering the existing heterogeneity, it could be inappropriate to choose a single global effect estimate to pool the data, and the summarized estimates in our study should be treated with caution [46]. Therefore, we performed stratified analyses to explain the possible sources of heterogeneity [47]. Additionally, some studies were case-control ones; these could lead to selection and recall bias.

Thirdly, there are several types of NSAID, which could have different effects on prostate cancer. However, most studies recruited in our study merely provided general data on NSAID intake rather than detailed information on specific dose and duration of use, which might bring about inaccurate estimates.

## Conclusions

We conducted a detailed meta-analysis for summarized OR estimates from researches focused on the association between NSAIDs intake and prostate cancer risk. Although we could not deny possible confounding factors, the results indicated a marginally inverse correlation between the intake of aspirin and prostate cancer risk. On the contrary, a positive relation between all-NSAID intake and prostate cancer was observed. Further research needs to be conducted to better clarify potential biological mechanisms.

## Abbreviations

CI: confidence interval
NSAID: nonsteroidal anti-inflammatory drug
OR: odds ratio.
